# The complex microbiome from native semen to embryo culture environment in human in vitro fertilization procedure

**DOI:** 10.1186/s12958-019-0562-z

**Published:** 2020-01-16

**Authors:** Jelena Štšepetova, Juliana Baranova, Jaak Simm, Ülle Parm, Tiiu Rööp, Sandra Sokmann, Paul Korrovits, Madis Jaagura, Karin Rosenstein, Andres Salumets, Reet Mändar

**Affiliations:** 1 Institute of Biomedicine and Translational Medicine, Department of Microbiology, University of Tartu, Ravila 19, 50411 Tartu, Estonia; 2 Competence Centre on Health Technologies, Tiigi 61B, 50410 Tartu, Estonia; 3 Department of Gene Technology, Tallinn University of Technology, Ehitajate tee 5, 19086 Tallinn, Estonia; 4 Tartu Health Care College, Nooruse 5, 50411 Tartu, Estonia; 5 Andrology Centre, Tartu University Hospital, L. Puusepa 1A, 50416 Tartu, Estonia; 6 NovaVita Clinic, Tammsaare tee 47, 11316 Tallinn, Estonia; 7 Institute of Biomedicine and Translational Medicine, University of Tartu, Ravila 19, 50411 Tartu, Estonia; 8 Institute of Clinical Medicine, Department of Obstetrics and Gynecology, University of Tartu, L. Puusepa 8, 50406 Tartu, Estonia; 9 Department of Obstetrics and Gynecology, University of Helsinki and Helsinki University Hospital, Haartmaninkatu 2, SF.00290 Helsinki, Finland

**Keywords:** Bacteria, Contamination, In vitro fertilization (IVF), Infertility, Sperm microbiota

## Abstract

**Background:**

Only a few microbial studies have conducted in IVF (in vitro fertilization), showing the high-variety bacterial contamination of IVF culture media to cause damage to or even loss of cultured oocytes and embryos. We aimed to determine the prevalence and counts of bacteria in IVF samples, and to associate them with clinical outcome.

**Methods:**

The studied samples from 50 infertile couples included: raw (*n* = 48), processed (*n* = 49) and incubated (*n* = 50) sperm samples, and IVF culture media (*n* = 50). The full microbiome was analyzed by 454 pyrosequencing and quantitative analysis by real-time quantitative PCR. Descriptive statistics, t-, Mann-Whitney tests and Spearman’s correlation were used for comparison of studied groups.

**Results:**

The study involved normozoospermic men. Normal vaginal microbiota was present in 72.0% of female partners, while intermediate microbiota and bacterial vaginosis were diagnosed in 12.0 and 16.0%, respectively. The decreasing bacterial loads were found in raw (35.5%), processed (12.0%) and sperm samples used for oocyte insemination (4.0%), and in 8.0% of IVF culture media. The most abundant genera of bacteria in native semen and IVF culture media were *Lactobacillus*, while in other samples *Alphaproteobacteria* prevailed. *Staphylococcus* sp. was found only in semen from patients with inflammation. Phylum *Bacteroidetes* was in negative correlation with sperm motility and *Alphaproteobacteria* with high-quality IVF embryos.

**Conclusion:**

Our study demonstrates that IVF does not occur in a sterile environment. The prevalent bacteria include classes *Bacilli* in raw semen and IVF culture media, *Clostridia* in processed and *Bacteroidia* in sperm samples used for insemination. The presence of *Staphylococcus* sp. and *Alphaproteobacteria* associated with clinical outcomes, like sperm and embryo quality.

## Background

Assisted reproductive technologies (ART) are the cornerstone of contemporary infertility treatment. Despite considerable progress made in ART, the implantation rate of replaced embryos remains low, and has been shown to depend on numerous clinical and laboratory factors. The success and failure in ART have largely been attributed to variables such as the patient’s age, weight, endometrial receptivity, and embryo quality and the transfer technique used. The viability of IVF embryos, in turn, depends on the composition of embryo culture media and physical environmental factors applied in embryo culture. At the same time bacterial contamination of gamete samples used in ART may impair the embryo culture environment, causing damage to or even loss of cultured oocytes and embryos [1].

Semen is not sterile [2] and may contain microorganisms even after processing for ART. Although most of the microorganisms detected in semen samples are non-pathogenic commensals or contaminants, their presence has a great significance on in vitro fertilization (IVF), a treatment in which the natural defense of the female genital tract is largely bypassed [3]. Therefore, different approaches have been proposed to reduce the microbial contamination and load in IVF culture media by improving semen preparation and embryo culture protocols. The majority of IVF laboratories use culture media containing antibiotics to minimize the risks of microbial growth. This has been a common practice since the first successful IVF treatment in 1978, when it was suggested that contamination during IVF procedure could negatively affect treatment outcome [4].

Nevertheless, microorganisms may colonize culture dishes with oocytes and embryos; most likely originating from semen samples, as follicular fluid samples are largely sterile and good laboratory practice of IVF eliminates the possibility that embryo culture media will become contaminated by microbes during the procedure. However, the exact frequency of these microbial contaminations is unknown due to low number of investigations [5]. Moreover, there is very little information available on how to handle embryos derived from culture dishes with obvious bacterial contamination. Therefore, the better understanding of whether seminal-derived bacteria have a negative impact on IVF conception could lead to the adoption of more efficacious interventions that can improve the pregnancy and delivery rate in assisted conception [6].

In the current study we aimed to determine the prevalence and counts of bacteria in native semen samples used for IVF, processed semen samples and IVF culture media, and to associate them with IVF embryo quality and pregnancy rate.

## Methods

### Ethical considerations

Participation in the study was voluntary. Informed written consent was obtained from the patients. The study was approved by the Ethics Review Committee on Human Research of the University of Tartu (Permission No. 193/T-16).

### Study group and lab standards

The study group included 50 infertile couples attending the Nova Vita clinic (Tallinn, Estonia) in 2012–2013 for IVF procedure. The average age of women and men were 33.4 ± 4.4 and 37.1 ± 6.3 years, respectively. Additional file 1: Tables S3, S4 provide the clinical and lifestyle background data for the study group. The patients had been suffering from infertility for at least 1 year being otherwise healthy. Only the couples undergoing IVF were recruited, while the couples requiring ICSI (intracytoplasmic sperm injection) were excluded.

Before IVF procedure, sexually transmitted infections were tested and treated whenever needed. Gram stained vaginal smears were microscopically examined to assess vaginal candidiasis as well as bacterial vaginosis according to standardized classification developed by Nugent [7]. Composite score was categorized into three categories, scores 0–3 being normal, 4–6 being intermediate, and 7–10 being definite bacterial vaginosis [7]. Inflammatory prostatitis was assessed by the neutrophil concentration in semen as described [8]. The involved IVF laboratory air quality corresponds to class D and cells were handled under the laminar where A class air quality is mandatory according to ISO 15189 standards. Air particle counting and microbiology measurements were done annually with no deviation.

### IVF, sample collection and processing

The patients underwent the standard ovarian stimulation with exogenous gonadotrophins promoting the multi-follicular development. Transvaginal ultrasonography-guided follicle aspiration was performed under short full anaesthesia. The follicles with size > 16 mm were aspirated, follicular fluid was evaluated under the stereomicroscope and oocyte-cumulus complexes were isolated and washed several times in clean culture medium (Origio Universal IVF media). Oocyte-cumulus complexes were incubated for 4 hours at 37 °C, 6% CO_2_ conditions until the planned insemination with washed semen.

Semen samples were obtained after 2–7 days of sexual abstinence. Before sample collection to sterile container men were asked to urinate and wash their glans penis with soap and warm water [2]. After ejaculation, the sperm sample was shortly (for maximum of 10 min) incubated at 37 °C and left for 25–45 min in room temperature for liquefaction. The semen analysis was performed according to WHO guidelines [9] (Additional file 1: Table S2). Thereafter the semen samples were processed using a 40–80% (2 ml + 2 ml) discontinuous gradient centrifugation method (PureSperm, Nidacon); to separate motile spermatozoa from non-living sperm cells, immotile spermatozoa and seminal plasma. 1–2 ml of the sperm ejaculate was layered over the gradient and centrifuged at 500 g for 20 min. After centrifugation, the supernatant was removed, and the sperm pellet was transferred to the clean 15 ml tube and resuspended in 5 ml of fresh medium (Sperm preparation medium, Origio). Thereafter a washing at 300 g for 10 min was performed, and the supernatant was discarded. The sperm pellet was gently transferred to new 5 ml tube and resuspended in 0.5–1 ml sperm-washing medium and incubated at room temperature for 1 h before oocyte insemination.

In normal IVF, oocytes were inseminated 4–5 h after follicular aspiration with ~ 150,000–200,000 progressively motile spermatozoa per 4–6 oocytes in 1 ml of culture media (Origio Universal IVF media). Fertilization was checked 16–18 h after insemination and normally fertilized oocytes with two pronuclei and polar bodies were further cultured in Origio ISM1 media for 24–48 h before being transferred or cryopreserved. Cleavage stage embryo quality was evaluated daily considering the number of blastomeres, the degree of fragmentation, and the uniformity of blastomeres. Embryos with better quality were selected for embryo transfer or cryopreserved for future use. The following embryo grading system was used: Grade 1 embryos have equal size and symmetrically located blastomeres with < 10% fragments; Grade 2 embryos have 10–25% fragmentation; Grade 3 and 4 embryos exhibit 25–50 and > 50% fragmentation, respectively. Uneven size of blastomeres, multinucleation and other abnormal features of the embryos downgrade the quality of the embryos. Both Grade 1 and 2 embryos are considered as good quality embryos and are preferred for transfer or cryopreservation, while Grade 3 embryos are classified as moderate quality embryos with lower chance of pregnancy [10].

**Fig. 1:**
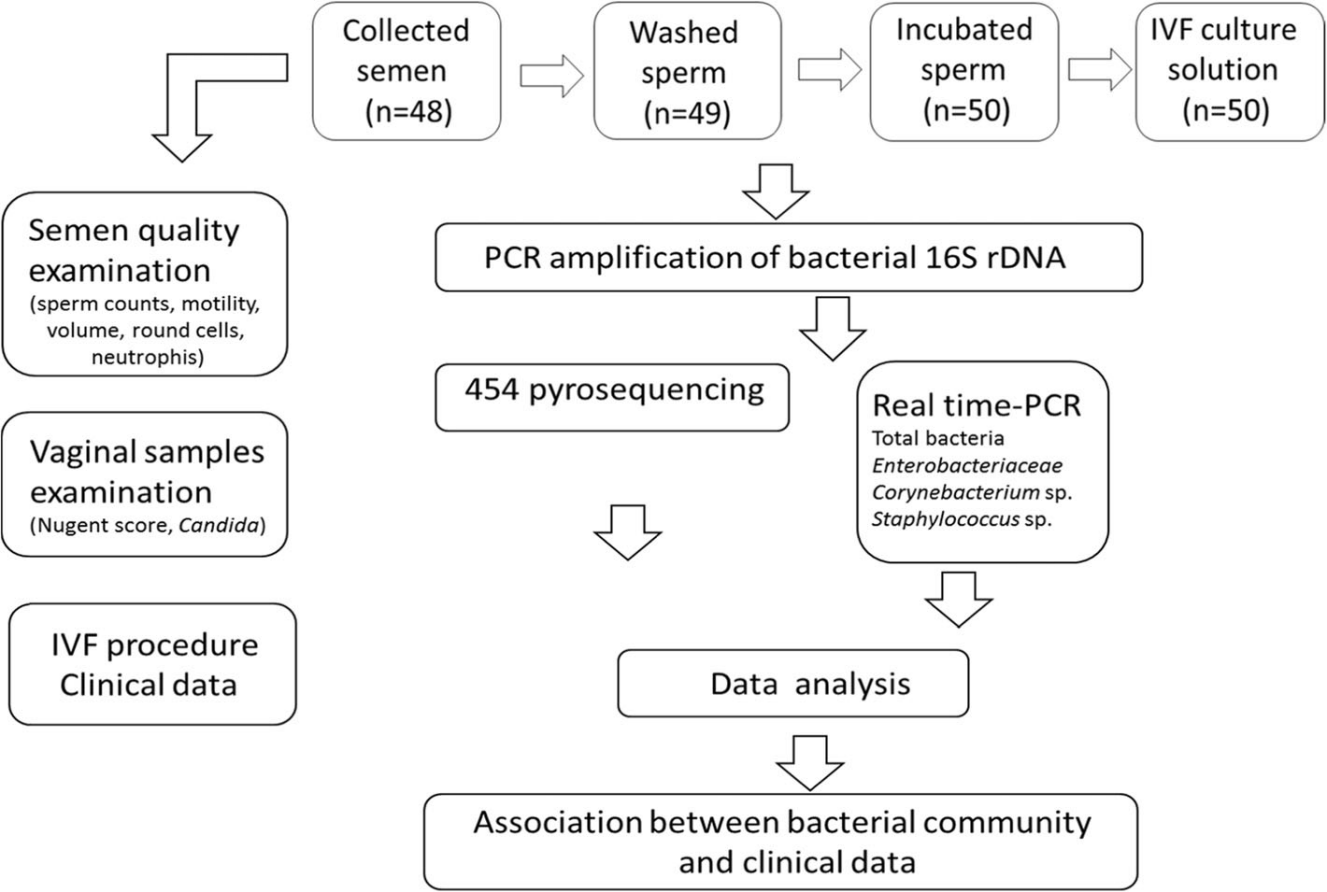
Overview of the study. Schematic information about samples and studies methods

Altogether 197 samples were available for analyses, including: 1) 0.2 ml fresh ejaculate samples (*n* = 48) frozen before processing; 2) 0.1–0.2 ml washed/prepared sperm samples prepared for oocyte insemination (*n* = 49) and frozen immediately after processing; 3) 0.1–0.4 ml of leftover semen suspensions (*n* = 50) used for oocyte insemination, but incubated overnight at 37 °C and 6% CO_2_, and frozen thereafter; and 4) 1–3 ml of collected IVF culture media (*n* = 50) where 4–6 oocytes have been incubated together with ~ 150,000–200,000 progressively motile spermatozoa for 16–18 h and frozen thereafter (Fig. 1). Samples were frozen at − 20 °C for further DNA extraction and microbiological studies.

Embryos were cultured usually for two or 3 days post fertilization and one or two embryos with better quality were selected for uterine transfer. A positive serum hCG test performed 2 weeks after embryo transfer confirmed biochemical pregnancy. The clinical pregnancy was documented by the presence of a positive fetal heart activity on transvaginal sonography at the sixth or seventh week of pregnancy.

### Molecular methods

Bacterial DNA of type strains was extracted using QiaAmp DNA mini kit (Qiagen, Hilden, Germany) according to manufacture instructions. DNA extraction from samples was performed using QIAamp DNA Blood Mini Kit (Qiagen) with some modifications. The sequencing of the DNA library was performed on the Roche 454 FLX next generation sequencing platform. Real-time PCR was applied to quantify the total counts of bacteria, *Enterobacteriaceae* group and indicator species *Staphylococcus* and *Corynebacterium*. The details of molecular methods are presented in Table 1 and Additional file 1: Table S1 [13, 16–18].

**Table 1:** Specific primers and probes used for 454 pyrosequencing and qPCR

Study methods	Target groups (amplicon length, T_m,_ assay)	Primers/Probes	Sequence (5′-3′)	References
454 pyrosequencing	16S rRNA gene	TIBACB-r^a^	TTGGCAGTCTCAG (NNNNNNNN)*AGAGTTTGATCCTGGCTCAG*	Fabrice et al. 2009 [11]
		TIBACA/TI357RA-f^a^	GTCTCCGACTCAG (NNNNNNNN)*CTGCTGCCTYCCGTA*	
		A	CCATCTCATCCCTGCGTGTCTCCGACTCAG	
		B	CCTATCCCCTGTGTGCCTTGGCAGTCTCAG	
Real-time PCR	Total bacteria (466 bp, 60 °C, TaqMan)	Univ-f Univ-r Univ (probe)	TGGAGCATGTGGTTTAATTCGA TGCGGGACTTAACCCAACA CACGAGCTGACGACA (AG)CCATGCA	Ott et al. 2004 [12]
	*Enterobacteriaceae* (195 bp, 58 °C, SYBR)	Eco1457-f Eco1652-r	CATTGACGTTACCCGCAGAAGAAGC CTCTACGAGACTCAAGCTTGC	Bartosch et al. 2004 [13]
	*Staphylococcus* sp. (560 bp, 62 °C, SYBR)	TstaG422 TstaG765	GGCCGTGTTGAACGTGGTCAAATCA TIACCATTTCAGTACCTTCTGGTAA	Martineau et al. 2001 [14]
	*Corynebacterium* sp*.* (516 bp, 60 °C, SYRR)	CF-1 CF-2	CGTAGGGTGCGAGCGTTGTCCG CGTTGCGGGACTTAACCCAACACT	Adderson et al. 2008 [15]

^a^In TIBACB and in TIBACA/TI357RA, A and B adapter sequences are underlined, the barcode is indicated by N-s in parentheses and specific primers’ sequences 8F and 357R are shown in italics. In reverse primer, the same barcode was incorporated to all samples

### Statistical analysis

The statistical analysis of clinical and qPCR data was performed using SIGMASTAT 2.0 (Systat Software, Chicago, USA) statistic software package. According to the data descriptive statistics, t-, Mann-Whitney tests and Spearman’s correlation were applied to compare the differences in microbiological indices. Statistically significant difference was considered if *P* < 0.05.

## Results

Altogether 50 couples attending IVF procedure participated in the study. For microbiological analyses two molecular approaches were combined, high-throughput sequencing that allows a global systemic view of the microbiome, and qPCR with specific primers that provide an accurate and sensitive method for quantification of individual bacteria in total bacterial count.

### Clinical indices

Clinical and lifestyle data of study subjects are presented in Additional file 1: Tables S2, S3 and S4. Semen volume and sperm motility and concentration were normal in all males (Additional file 1: Table S2). According to the WHO threshold value [19], the subset of men with increased neutrophil concentration in semen was 20.0% (10/50). The sperm concentrations were higher before washing in comparison to after washing and sperm suspension used for insemination (*p* < 0.001, both). In contrast, the sperm motility (A + B) were increased after sperm washing (*p* < 0.001), where A and B are progressively moving sperm cells.

According to Nugent score data, normal vaginal microbiota was present in 72.0% (36/50) of women; additionally, intermediate microbiota and bacterial vaginosis were diagnosed in 12.0% (6.0/50) and 16.0% (8.0/50) of women, respectively (Additional file 1: Table S2). Biochemical pregnancy after IVF embryo transfer was recorded in 36.0% (18/50) of the couples, while ultrasound scan confirmed the clinical pregnancy in 28.0% (14/50) of the cases.

### Microbiome of samples used for IVF procedure

We applied the pyrosequencing of 16S *r*RNA V2-V3 region to reveal the full microbiome of the investigated samples. Among all 197 samples, 35.5% (17/48) of raw semen, 12.0% (6.0/49) of washed sperm, 4.0% (2.0/50) of incubated semen samples, and 8.0% (4.0/50) of IVF culture media were positive by sequencing method. The number of sequences decreased in studied samples during sperm treatment (Table 2).

**Table 2:** Average number (±SD) of sequences, phylotypes abundance (OTUs) and Shannon ‘H’ diversity index in the studied samples

Samples	Number of sequences (gene copies/μl)	Phylotype abundance (OTUs)	Shannon ‘H’ index (diversity)
Semen	7911 ± 3562^a,b,c^	81.5 ± 44.5	33.6 ± 16.5
Washed sperm	4100 ± 3931^a^	64.5 ± 38.5	35.6 ± 7.9
Incubated sperm	1692 ± 1296^b^	30.5 ± 12.0	24.4 ± 4.3
IVF culture media	2572 ± 1080^c^	36.2 ± 5.7	30.6 ± 6.9

^a^*p* = 0.038

^b^*p* = 0.027

^c^*p* = 0.008

In total, 188,983 sequences were obtained, with an average 7911 ± 3562 reads for each of the raw semen sample, an average of 4100 ± 3931 and 1692 ± 1296 reads for washed and incubated sperm samples, respectively; as well as an average of 2572 ± 1080 reads for IVF insemination media. The phylotype abundance and Shannon ‘H’ diversity index were also higher in semen and washed sperm than the incubated sperm and IVF culture media though the differences were slightly over significance level.

**Fig. 2:**
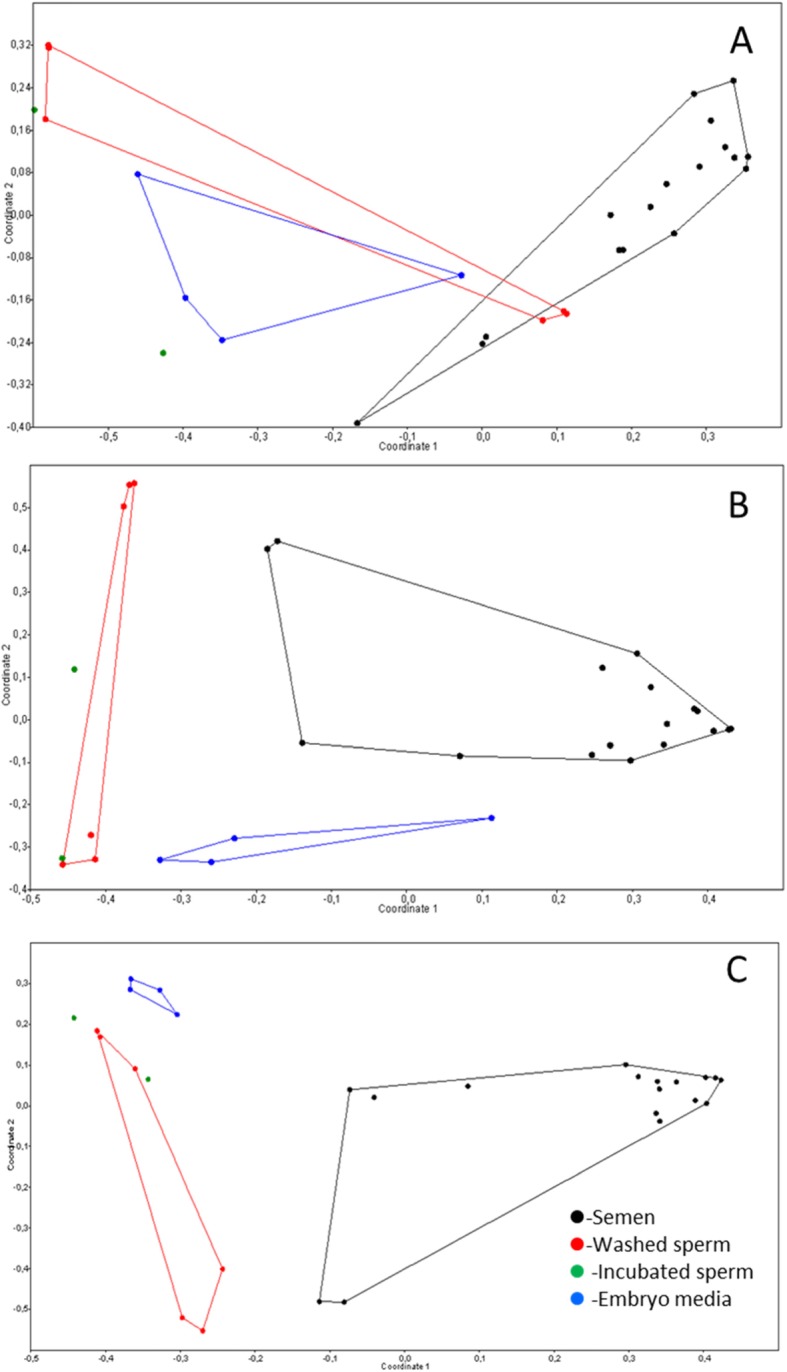
Principal coordinate analysis (PCoA) of bacterial communities in studied samples based on (**a**) phylum, (**b**) classes and (**c**) genera levels. A principal coordinate analysis plot demonstrates different clustering of different specimens (semen, washed sperm, incubated sperm and IVF culture solution)

**Fig. 3:**
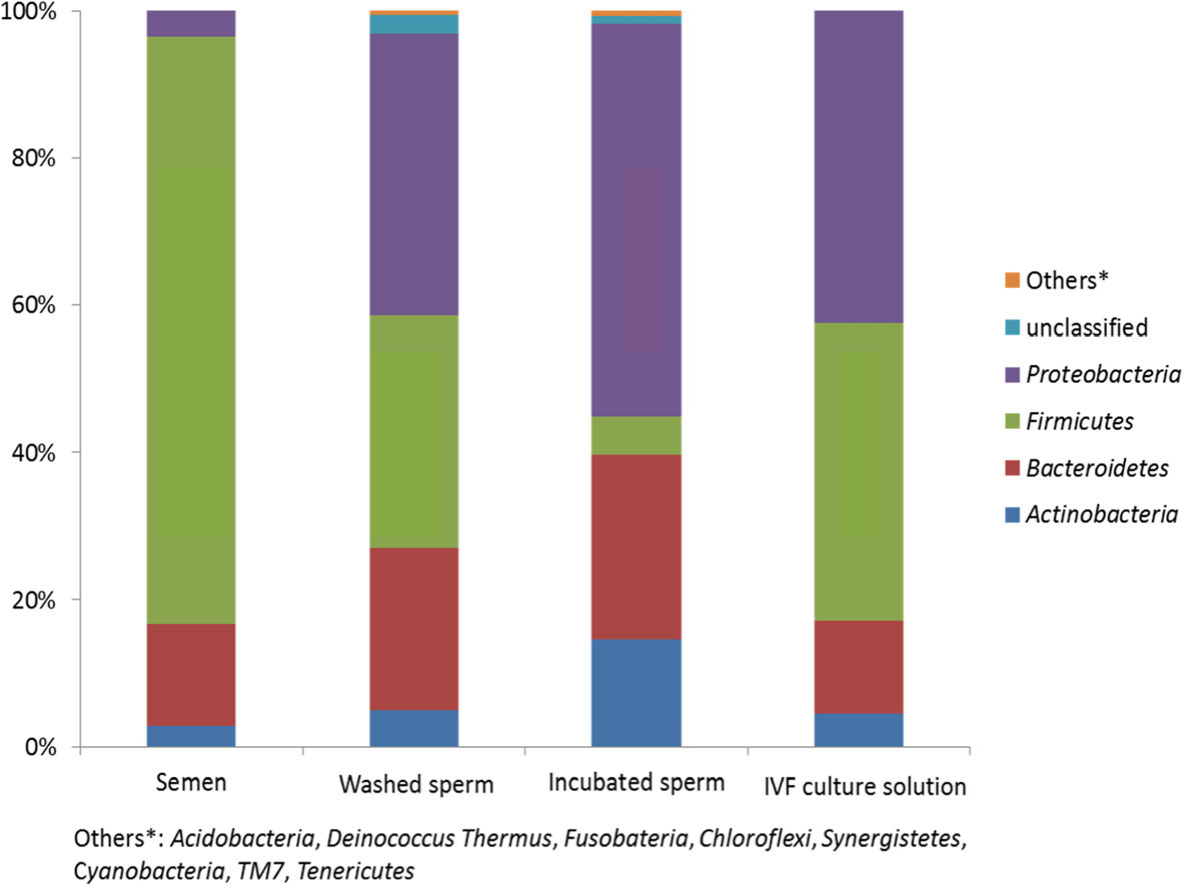
Relative abundance of different bacterial phyla in microbial communities of different samples. Bar charts showing mean values of 4 most abundant phyla in semen, washed and incubated sperm and IVF culture solution. Others: *Acidobacteria, Deinococcus Thermus, Fusobacteria, Chloroflexi, Synergistetes, Cyanobacteria,* TM7 and *Tenericutes.* Phylum *Firmicutes* displayed the highest relative abundance in semen. The processed/washed sperm solution displayed more diverse composition of bacteria, in addition to *Firmicutes* also *Proteobacteria* and *Bacteroidetes* displayed remarkable proportions. Almost half of the bacteria in incubated semen and in IVF culture media were represented by *Proteobacteria*

A principal coordinate analysis (PCoA) plot based on different taxonomic levels (phylum, classes and genera) was constructed to assess the relationships between the community structures of studied samples. Figure 2 showed that the microbiota of different studied samples clustered separately as expected. In native semen the *phylum* *Firmicutes* displayed the highest relative abundance (median 91.5%) (Fig. 3, Additional file 1: Table S5). The processed/washed sperm solution displayed more diverse composition of bacteria, in addition to *Firmicutes* also *Proteobacteria* and *Bacteroidetes* displayed remarkable proportions (medians 19.6 to 36.4%). Almost half of the bacteria in incubated semen and in IVF culture media were represented by *Proteobacteria*.

**Fig. 4:**
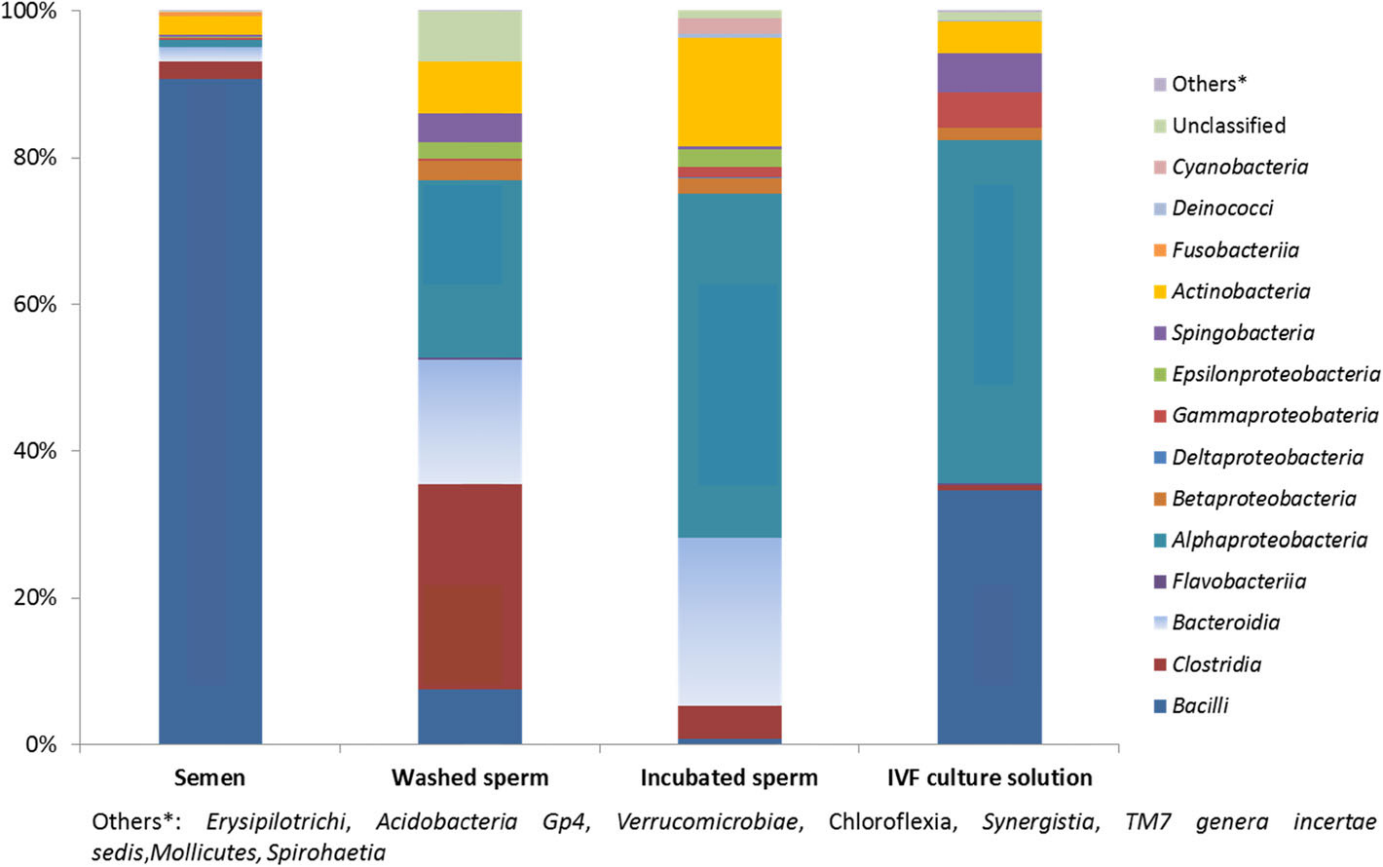
Relative abundance of different bacterial classes in microbial communities of different samples. Bar charts showing mean values of most abundant classes in semen, washed and incubated sperm and IVF culture solution. Others: *Erysipilotrichi, Acidobacteria Gp4, Verrucomicrobiae, Chloroflexia, Synergistia, TM7 eneta incertae sedis, Mollicutes* and *Spirohaetia. Bacilli* displayed the highest relative abundance in sperm before washing and in IVF culture media; *Clostridia* in washed sperm and *Bacteroidia* in both washed and incubated sperm. *Alphaproteobacteria* displayed high proportions in incubated sperm and IVF culture media

On class level, *Bacilli* displayed the highest relative abundance in sperm before washing (85.7%) and in IVF culture media (32.7%); *Clostridia* (20.6%) in washed sperm and *Bacteroidia* in both washed and incubated sperm (12.6 and 22.4%) (Fig. 4, Additional file 1: Table S6). *Alphaproteobacteria* displayed high proportions in incubated sperm and IVF culture media (45.7 and 44.1%).

**Fig. 5:**
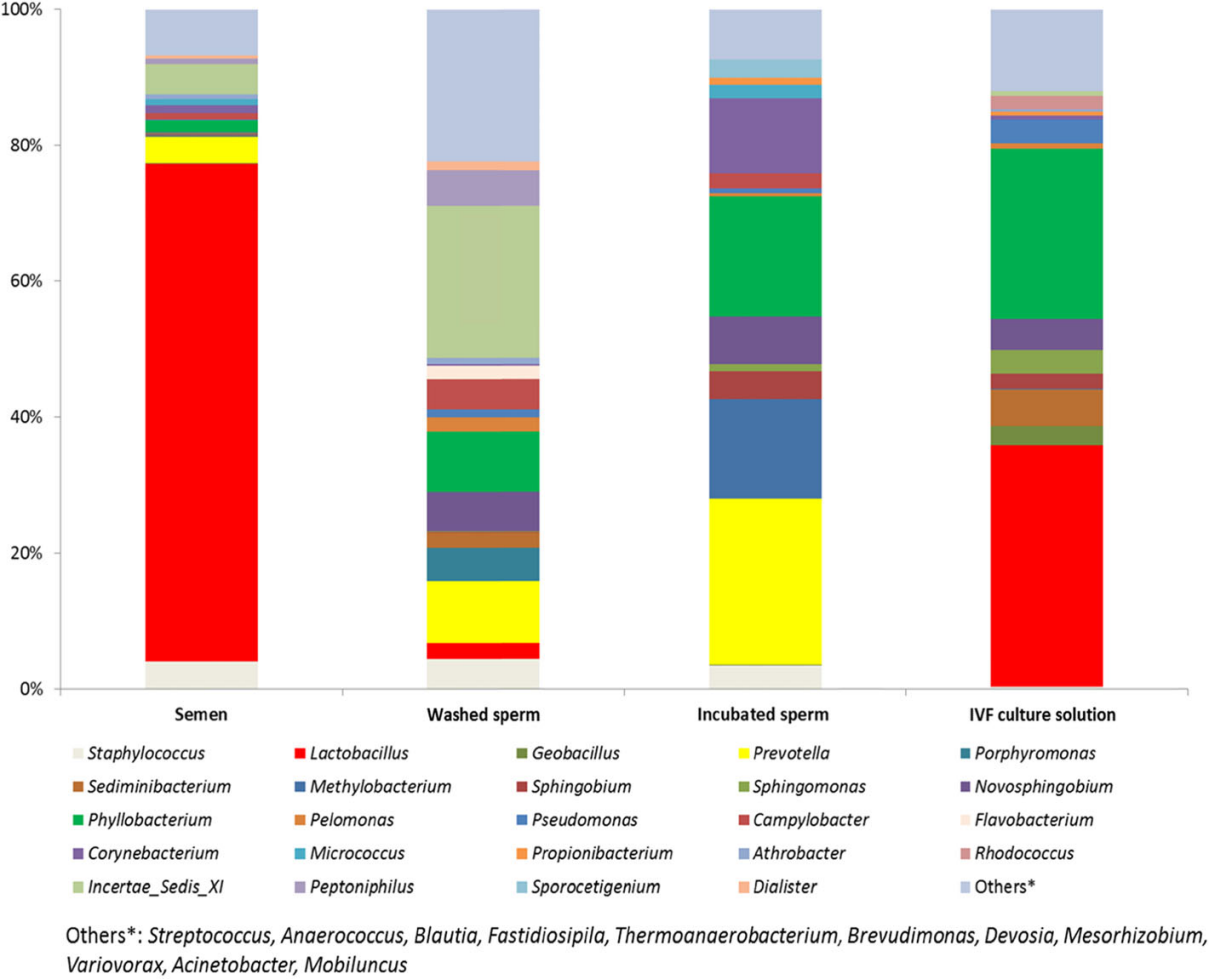
Relative abundance of most frequent bacterial genera of microbial communities of different samples. Bar charts showing mean values of most abundant genera in semen washed and incubated sperm and IVF culture solution. Others: *Streptococcus, Anaerococcus, Blautia, Fastidiosipila, Thermoanaerobacterium, Brevudimonas, Devosia, Msorhizobium, Variovorax, Acinetobacter* and *Mobiluncus.* The most abundant genera of bacteria in the sperm before washing and IVF culture solution were *Lactobacillus*, followed by *Incertae sedis* XI, *Staphylococcus* and *Prevotella* in raw semen samples

The most abundant genera of bacteria in the sperm before washing and IVF culture solution were *Lactobacillus* (73.3 and 35.5%, respectively), followed by *Incertae sedis* XI (4.5%), *Staphylococcus* (4%) and *Prevotella* (3.9%) in raw semen samples, while in other samples more heterogenous microbial composition was noted (Fig. 5, Additional file 1: Table S7).

### Prevalence of common aerobic bacteria in IVF samples as revealed by qPCR method

**Fig. 6:**
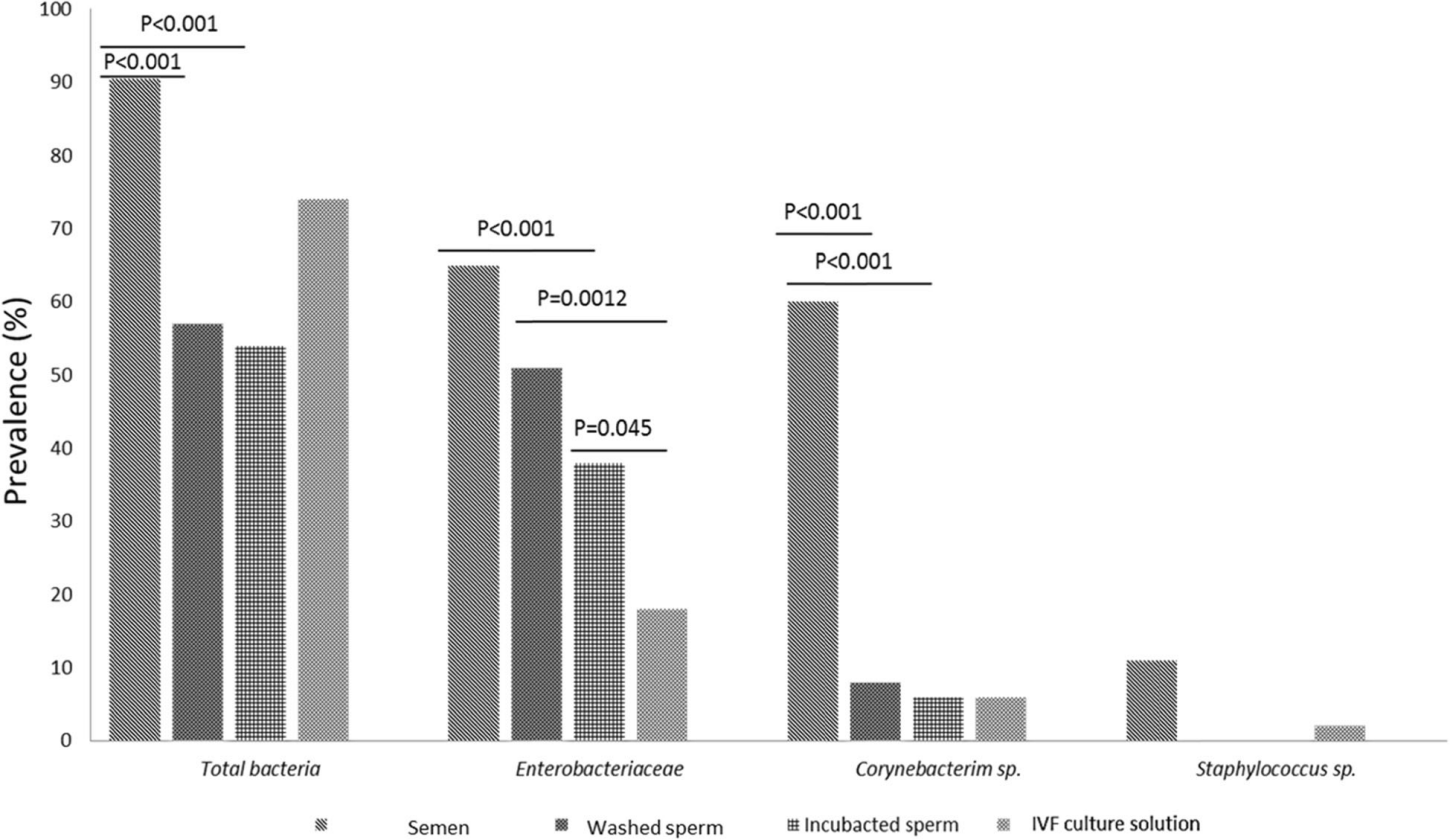
The prevalence (%) of total and three common groups of bacteria *Enterobacteriaceae, Corynebacterium* sp. and *Staphylococcus* sp. according to qPCR in study samples. The prevalence of bacteria in the studied sperm samples significantly decreased after washing and incubation. The prevalence of *Enterobacteriaceae* was lower in IVF culture media than in washed and incubated semen

We additionally applied qPCR to detect prevalence and concentration of total bacteria as well as three common groups of bacteria in male semen – *Enterobacteriaceae, Corynebacterium* sp*.* and *Staphylococcus* sp*.* The prevalence of bacteria in the studied sperm samples significantly decreased after washing and incubation (Fig. 6); while the mean total counts of bacteria decreased during all treated procedures (Table 3). The prevalence of *Enterobacteriaceae* was lower in IVF culture media than in washed and incubated sperm (Fig. 6), while the counts were lowest in incubated sperm than in raw and washed sperm (Table 3). The counts of *Corynebacterium* sp. were higher in raw semen in comparison to both washed and incubated sperm as well as IVF insemination media (Table 3).

**Table 3:** The counts (log^10^ plasmid gene copies/ml sperm; mean ± SD) of total bacteria and three common groups of bacteria, *Enterobacteriaceae, Corynebacterium* sp. and *Staphylococcus* sp., in study samples as revealed by qPCR

Bacterial groups	Semen	Washed sperm	Incubated sperm	IVF culture media	*p*
Total bacteria	5.79 ± 0.44^a,b,c^	5.17 ± 0.53^a^	4.92 ± 0.36^b^	4.85 ± 0.28^c^	^a,b,c^ *p* < 0.001
*Enterobacteriaceae*	4.67 ± 0.89^a,b^	3.92 ± 0.26^a,c,d^	3.43 ± 0.31^b,d^	3.95 ± 0.56^c^	^a^ *p* < 0.005 ^b,c^ *p* < 0.001 ^d^ *p* = 0.008
*Corynebacterium* sp.	4.51 ± 0.67^a,b,c^	3.78 ± 1.01^a^	4.04 ± 0.35^b^	3.36 ± 0.49^c^	^a,b,c^ *p* < 0.001
*Staphylococcus* sp.	3.55 ± 0.33	0	0	2.58	NS

^a,b,c,d^ Indicate the compared pairs of values that resulted in statistically significant differences (*p* value less than 0.05, given in the last column)

### Associations between bacteria detected both by sequencing and qPCR, and clinical data

Positive correlations between neutrophils and certain bacteria in raw semen (genus *Staphylococcus,* classes *Erysipelotrichia* and *Bacteroidia*) were found (Table 4).

**Table 4:** Spearman’s rank-order correlation between bacteria presented in raw semen and washed sperm (*) detected by pyrosequencing (454), qPCR and clinical data

Bacteria	Clinical data	R	*P*
Presence of *Bacteroidia* (454, class)	Neutrophils	0.673	0.0028
Presence of *Erysipelotrichia* (454, class)		0.523	0.0309
*Erysipelotrichia* (454, OTU numbers)		0.636	0.006
Counts of *Staphylococcus* spp. (qPCR)		0.448	0.002
Total OTU numbers (454, phylum)	Sperm motility	−0.472	0.0551
*Bacteroidetes* (454, phylum, OTU numbers)		−0.518	0.0328
*Bacteroidia* (454, class, OTU numbers)		−0.498	0.0411
*Proteobacteria* (454, phylum, OTU numbers)		−0.661	0.0036
*Alphaproteobacteria* (454, class, OTU numbers)		−0.471	0.0551
*Sphingobacteria* (454, class, OTU numbers)		−0.635	0.0006
*Alphaproteobacteria* (454, OTU numbers)*	Embryo quality (Grade 1, 2, 3)	0.413	0.0260

*Staphylococcus* sp. was detected only in semen samples of patients with inflammation.

At the same time the class *Bacteroidia,* and the whole phylum *Bacteroidetes* of raw semen were in negative correlation with sperm motility, like also some other bacteria – *Proteobacteria* (phylum), *Alphaproteobacteria* (class), and *Sphingobacteria* (class).

**Fig. 7:**
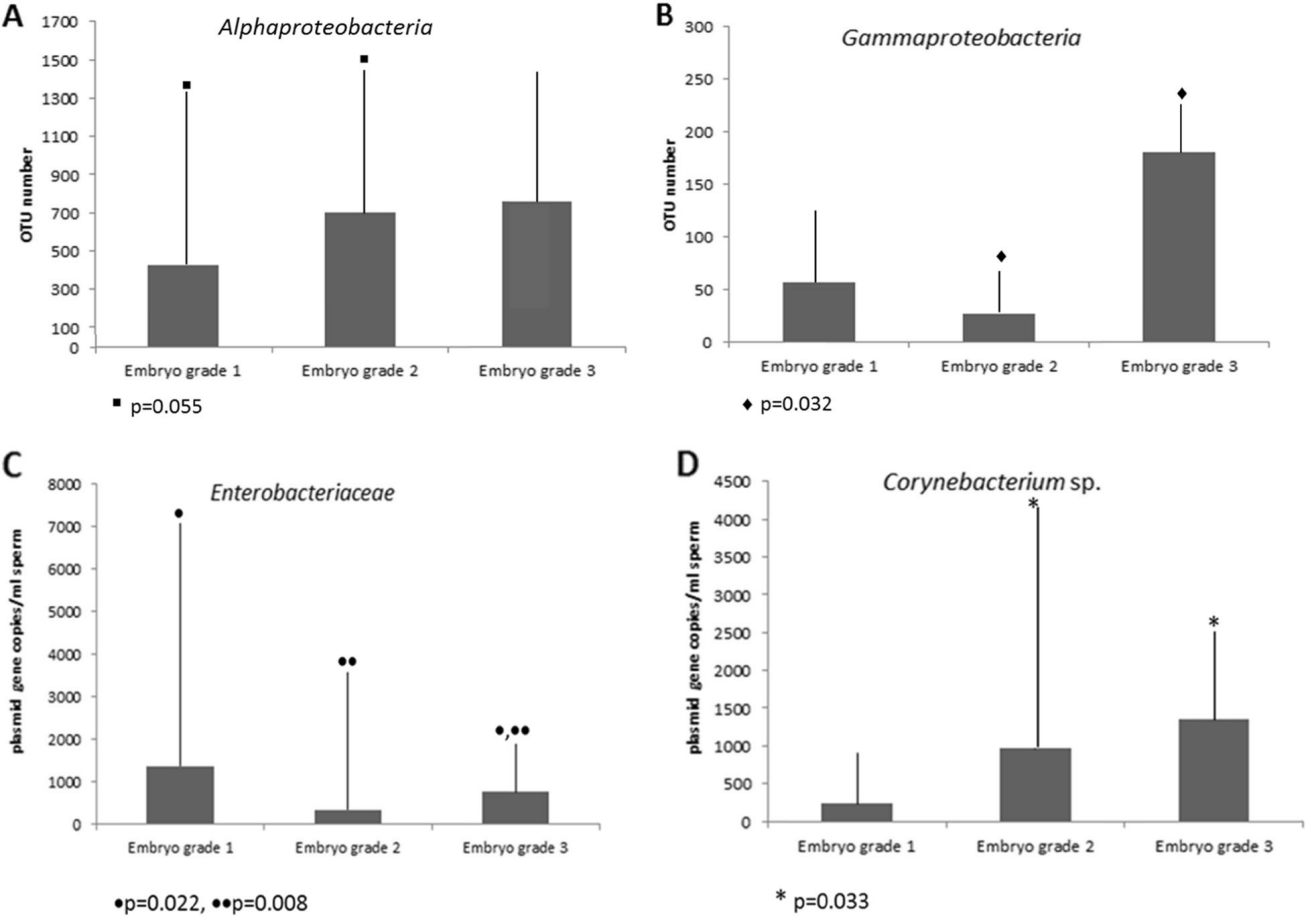
The differences in the counts of *Alphaproteobacteria* (**a**) and *Gammaproteobacteria* (**b**); *Enterobacteriaceae* (qPCR) (**c**) and *Corynebacterium* sp. (qPCR) (**d**) in solutions with different embryo grades. The higher counts of *Alphaproteobacteria* (**a**) and *Gammaproteobacteria* (**b**) (454 pyrosequencing, mean + SD, OTU), in washed sperm, and *Corynebacterium* sp*.* (**d**) (qPCR, mean + SD, plasmid gene copies/ml sperm) in raw semen samples were found in patients with lower embryo quality (**a**, **b**, **d**). The mean proportion of *Enterobacteriaceae* (**c**) group (qPCR) in raw semen was higher in couples with better embryo quality (**c**)

The positive correlation between *Alphaproteobacteria* (454 pyrosequencing) in washed sperm and low-quality embryos was found (Table 4). Additionally, the higher counts of *Alphaproteobacteria* and *Gammaproteobacteria* (454 pyrosequencing) in washed sperm, and *Corynebacterium* sp*.* (qPCR) in raw semen samples were found in patients with lower embryo quality (Fig. 7a, b, d). However, the mean proportion of *Enterobacteriaceae* group in raw semen was higher in couples with better embryo quality (Fig. 7c). No correlation between the prevalence or counts of bacteria presented in IVF culture media and pregnancy results was found.

## Discussion

This study revealed the qualitative and quantitative bacterial composition of the samples used in IVF. We found that there are considerable bacterial changes in IVF samples with prevalence of classes *Bacilli* in raw semen and embryo culture media, *Clostridia* in washed sperm, *Bacteroidia* in incubated sperm and *Alphaproteobacteria* in incubated sperm and IVF culture media. The associations between certain clinical data (such as increased counts of neutrophils, sperm motility, embryo quality) and presence of some bacterial phyla and genera (*Bacteroidetes, Proteobacteria, Staphylococcus*, *Corynebacterium* spp.) were also found. Although our study is not the first research project describing the presence of some groups of microbes in raw and processed sperm and IVF culture media [2, 20, 21], our study is the first one giving a deep assessment of bacterial composition of IVF culture media based on 16S rRNA gene fragments (454 sequencing platform) to help monitor IVF culture conditions.

The goal of embryo culture in IVF is to keep gametes and embryos in as similar condition to their native environment. For that in IVF laboratory, the maintenance of gametes and embryos requires stringent culture conditions. A high standard of hygiene, cleaning and waste disposal must be followed in order to avoid infection of medical staff and patients, and contamination of the culture dishes and equipment. Every step in the laboratory procedures and manipulations must be carried out with a rigorous discipline of aseptic techniques [22]. Therefore, the sterile culture conditions should be pursued in the conditions, where semen samples and follicular fluid samples – obtained via transvaginal ultrasound-guided aspiration, are believed to contain polymicrobial communities. Indeed, the presence of bacteria in environment and patients’ bodies, like semen and follicular fluid samples, and cervical regions passed in egg retrieval and embryo transfer, have been associated with adverse pregnancy outcomes in IVF [23]. Similarly, a small number of researchers have reported isolating microorganisms from IVF culture media [24, 25]. Semen, technician contamination, for example from oil overlaying human embryo culture, is the most often quoted sources of contamination. The most common species identified are *Escherichia coli*, *Aspergillus*, *Candida albicans* and Gram-negative cocci [23].

We found bacterial load of embryo media in around 8% of the samples by 454 sequencing and more than 70% by real-time PCR method. Previously, Kastrop et al. examined > 14,000 and Ben-Chetrir et al. > 700 IVF cycles by cultivation and found that in both studies 0.7% of IVF cycles had isolated microorganisms [5, 26]. The differences in results may be explained by methods used for bacteria examination. In our study the lower limit of PCR amplicons for 454 sequencing was 0.5 ng/μl but for real-time PCR we used DNA of all 197 isolated samples. Additionally, qPCR specific primers for *Enterobacteriaceae* group (*Gammaproteobacteria*) may amplify some other bacterial species such as *Moellerella*, *Morganella*, *Proteus*, *Leminorella* and etc. [13] that was not found by 454 pyrosequencing. It may equally be explained by the fact that the qPCR used in the current study is more sensitive for specific groups of bacteria that agrees with the study of Al-Mously et al. [27].

The microbiome of semen has been studied mostly in connection with male infertility or prostatitis [28–30]. We found that the predominant bacterial genera in semen samples were *Lactobacillus*, *Incertae sedis XI*, *Staphylococcus*, *Prevotella*, *Phyllobacterium* and *Corynebacterium*. Previously, the high abundance of *Lactobacillus* in semen was also published [2, 21, 29]. Most abundant genera presented in semen by Hou et al. study were also identified abundant in our data, such as *Lactobacillus*, *Prevotella*, *Corynebacterium*, *Staphylococcus* and *Veillonella* [31]. Semen quality in *Lactobacillus*-predominant semen samples is higher than in the case of the other community types, as lactobacilli prevent sperm lipid peroxidation, thus preserving sperm motility and viability [28]. Some authors indicated that Gram-positive bacteria such as *Lactobacillus* and *Corynebacterium*, might protect against the negative influence of Gram-negative bacteria such as *Prevotella*, *Aggregatibacter* and *Pseudomonas* [21]. *Prevotella* is a genus of Gram-negative anaerobic bacteria [32], that is a member of both semen and vaginal microbial communities while its increased counts have been described in patients with low-quality semen [21, 33]. The clinical significance of strict anaerobes in sperm samples is a subject of dispute. Anaerobic bacteria are not routinely sought in sperm samples, because they are fastidious to cultivate. In fact, by using molecular methods Kiessling et al. detected and identified many anaerobes in the semen of men undergoing fertility evaluation [34]. Our study demonstrated correlation between presences of gram-negative bacteria (*Bacteroidia, Sphingobacteria* (class), *Proteobacteria* (phylum), *Alphaproteobacteria* (class)) with sperm motility. The gram-negative bacteria contain in their cell walls lipopolysaccharide that associated with more pro-inflammatory and oxidant environment and due to these mechnanism may disrupts sperm motility [35].

There is no data about the presence of *Incertae sedis XI* in semen samples*.* Previous publications indicated that *Clostridiales Family XI Incertae Sedis* bacteria are enriched in the colons of healthy adults and are also found on skin and genitals of women suffering from bacterial vaginosis [36–38].

Similar to semen*, Lactobacillus* genus was also dominating in embryo culture media. Next generation sequencing revealed that *Lactobacillus* sp. are present in endometrial and ovarian follicular microbiome [25, 39]. The authors associated it with embryo development, and difference in the microbiome between the left and right ovaries, which was attributed to differences in haematogenous spread, was also demonstrated [40]. In contrast, the presence of some other species, such as *Propionibacterium* and *Actinomyces* among others, has been associated with adverse IVF outcomes. In addition, *E. coli* and *Streptococcus* spp. in follicular fluid might inhibit follicle-stimulating hormone (FSH) from binding to its receptor on granulosa cells [41, 42]. To conclude, the follicular fluid bacteria have been associated with both positive and negative IVF outcomes [25, 40]. In our study, the presence of bacteria in IVF culture media did not influence the pregnancy rate. Also, we were unable to determine the origin of the microbiota in embryo culture media.

Since incubation temperature is a determining factor for bacterial growth, incubation of IVF media at 37 °C could influence the bacterial growth and activity. We found that washed and incubated sperm samples had quite heterogenous microbial composition with prevalence of genera *Prevotella* and *Staphylococcus,* and class *Alphaproteobacteria.* Interestingly, *Alphaproteobacteria* was the most prevailed class of bacteria in processed sperm samples without and with further incubation, including the highest prevalence of genera *Phyllobacterium* in all treated sperm samples as well as *Methylobacterium* in incubated sperm and *Novosphingobium* in washed sperm and embryo culture media. Presence of these genera in IVF culture media was not previously published. At the same time previous studies have indicated that coliform bacteria, including *E. coli* that belong to *Alphaproteobacteria* were found in higher concentration in semen and media used during IVF procedure [5, 21, 43]. Kala et al. demonstrated that inoculation of *E. coli* caused adhesion to the sperm membrane and subsequent destruction leading to reduced motility and viability in washed samples [44]. Presence of *S. aureus* and *E. coli* may induce apoptosis in human sperm with two possible putative mechanisms: a direct cytotoxic activity of bacterial toxins and the contact with pili and flagella [28].

The majority of IVF laboratories use culture media containing antibiotics to avoid the risks of microbial growth during IFV procedure. The most commonly used antibiotics are penicillin (β-lactam), streptomycin and gentamicin [45]. In our study, both semen incubation media and embryo culture media were supplemented with gentamicin sulphate. Gentamicin is a broad-spectrum bactericidal agent of aminoglycoside group that is effective against Gram-positive and Gram-negative aerobic bacteria. Gentamicin binds to four nucleotides of 16S rRNA and a single amino acid of protein S12. This leads to interference with the initiation complex and misreading of mRNA so that incorrect amino acids are inserted into the polypeptide leading to non-functional or toxic peptides and the breakup of polysomes into non-functional monosomes. Though the counts of *Enterobacteriaceae* decreased with treatment (qPCR), the abundance of some species (*Methylobacterium, Phyllobacterium*) belonging to *Alphaproteobacteria* classes increased. Some of these bacteria species may be resistant to gentamicin. The resistance of *E. coli* to both penicillin and streptomycin in culture media has been reported previously [5, 24]. Although, in the study of 70 bacterial strains isolated from contaminated culture media were subsequently found to be sensitive to gentamicin, we may support the view that antimicrobials in the culture media probably provide little inhibition to the potentially large numbers of bacteria, including anaerobic bacteria. Moreover, it has been shown that aminoglycosides have toxic effects on sperm motility [46]. A review analyzing randomized controlled trials investigating the effect of antibiotics on embryo transfer concluded that the administration of amoxycillin and clavulanic acid prior to embryo transfer reduced the upper genital tract microbial contamination but did not influence clinical pregnancy rates [47]. Furthermore, there are no data about randomised controlled trials to support or refute other antibiotic regimens in this setting [47, 48].

Our findings showed that the simple presence of bacteria might alter the sperm quality. In the present study the counts of *Staphylococcus* sp*.* were correlated with presence of neutrophils in semen. Previously, Moretti et al. demonstrated the sperm concentration and the percentage of progressive motility were significantly decreased in sperm samples containing *S. epidermidis, S. aureus* and *E. coli* [28]. In addition, we found that counts of *Alphaproteobacteria* and *Enterobacteriaceae* may influence embryo quality. In accordance with our results, it has been previously indicated that if the embryo culture dishes are contaminated with bacteria the quality of the developing embryos is poor [5].

Our study has some limitations. First, the number of samples was moderate. In addition, qPCR did not cover wide spectrum of bacteria.

## Conclusion

In conclusion, our study demonstrated that IVF does not occur in a sterile environment. The prevalence and counts of bacteria in the IVF procedure decrease during the semen treatment. We demonstrated the prevalence of classes *Bacilli* (*Lactobacillus* genera) in raw semen and IVF culture media, *Clostridia* in washed sperm and *Bacteroidia* in incubated sperm samples. The presence of *Staphylococcus* sp. and *Alphaproteobacteria* are associated with clinical indicators such as sperm and embryo quality. Therefore, future research should focus on the methods aiding to reduce the adverse impact of these microorganisms on IVF embryo development and helping to avoid IVF failure.

## Supplementary information

**Additional file 1: Table S1.** Details of molecular methods. **Table S2.** Overview of the semen and vaginal samples of the study subjects. **Table S3.** Overview of general and reproductive health of female partners. **Table S4.** Overview of general and reproductive health of male partners. **Table S5.** Relative abundance (%) of different bacterial phyla in microbial communities of different samples (mean±SD). **Table S6.** Relative abundance (%) of different bacterial classes in microbial communities of different samples (mean±SD). **Table S7.** Relative abundance (%) of most frequent bacterial genera of microbial communities of different samples (mean±SD).

## Data Availability

The datasets used and/or analyzed during the current study available from the corresponding. author on reasonable request.

## Abbreviations

ART: Assisted reproductive technologies
FSH: Follicle-stimulating hormone.
hCG test: Human chorionic gonadotropin test
ICSI: Intracytoplasmic sperm injection
IVF: In vitro fertilization
PCoA: Principal coordinate analysis
qPCR: Quantitative polymerase chain reaction
