# Supplementary material for: The complex microbiome from native semen to embryo culture environment in human in vitro fertilization procedure
Source: Reprod Biol Endocrinol. 2020 Jan 16;18:3. doi: 10.1186/s12958-019-0562-z (PMC6966866; doi:10.1186/s12958-019-0562-z)
Supplement: Supplementary file 1 — Additional file 1: Table S1. Details of molecular methods. Table S2. Overview of the semen and vaginal samples of the study subjects. Table S3. Overview of general and reproductive health of female partners. Table S4. Overview of general and reproductive health of male partners. Table S5. Relative abundance (%) of different bacterial phyla in microbial communities of different samples (mean±SD). Table S6. Relative abundance (%) of different bacterial classes in microbial communities of different samples (mean±SD). Table S7. Relative abundance (%) of most frequent bacterial genera of microbial communities of different samples (mean±SD). [file 12958_2019_562_MOESM1_ESM.docx]

**Additional file 1**

**Supplementary Table S1** Details of molecular methods

| ***Bacterial strains and growth conditions*** |
| --- |
| Bacterial type strains *Escherichia coli* ATCC 700336, *Staphylococcus epidermidis* ATCC 12228 from the American Type Culture Collection (ATCC) and sperm bacterial isolate, identified by 16S rRNA sequencing (similarity with BLAST 100%) as *Corynebacterium seminale* were used to evaluate the specificity of qPCR primer sets. |
| ***DNA extraction from bacterial cultures and sample*** |
| Bacterial DNA of type strains was extracted using QiaAmp DNA mini kit (Qiagen, Hilden, Germany) according to manufacture instructions. DNA extraction from samples was performed using QIAamp DNA Blood Mini Kit (Qiagen) with some modifications. 180 µl of raw semen were added into 10 ml buffer (150 mM sodium chloride (NaCl), 10 mM EDTA pH=8.0), vortexed for 10 sec at full speed and centrifuged for 10 min at 4,000 rpm. Bacterial pellets were resuspended in 300 µl buffer (100 mM Tris-Cl pH=8.0, 10 mM EDTA, 500mM NaCl, 1% sodium dodecyl sulphate (SDS), 2% mercaptoethanol). 120 µl Proteinase K was added into pellets and incubated 4 h at 55ºC on a rocking platform. In addition, 400 µl Buffer AL and 400 µl ethanol was added to the sample, and mixed by vortexing. From this point, the extraction proceeded following the standard protocol (Qiagen).  All other samples were transferred to a 1.5 ml microcentrifuge tubes, and centrifuged at 13,000 rpm for 2 min to pellet the bacteria. Bacterial pellets were suspended in 180 µl of lysis buffer (20mM Tris-HCl, 2 mM EDTA, 1.2% TritonX-100, 20 mg/ml lysozyme, pH=8.0) and incubated for at least 30 min at 37°C. In addition, 25 µl Proteinase K and 200 µl Buffer AL were added to the sample and mixed by vortexing. Each suspension was incubated at 70°C for 30 min. The protocol was then continued as described by manufacturer (Qiagen). Extracted DNA was quantified using NanoDrop^TM^ 1000 Spectrophotometer 1.0 (Nano drop Technologies, Inc., USA) at 260 nm. The DNA extracts were stored at −20°C for further analysis. |
| **454 pyrosequencing of 16S *rRNA* gene** |
| The amplification of 16S rRNA V2-V3 region was performed with primers that included 454 specific adaptor sequences at 5’-end following the 8-bp barcode marked as Ns (unique sequence tag to barcode each sample) and universal TIBACB-r and TUBACA/TI357RA-f primers (Table 1). PCR was carried out in two sequential reactions of which first was targeted PCR with region specific primers, multiplex identifying barcodes and partial adapters for sequencing, and second PCR was used to complete sequencing adapters. 3 µl of stock DNA samples was used in the first PCR and 3 µl of 10-fold dilution of amplicon was used in the second PCR reaction. The total reaction volume of PCR was 30 µl, and contained primers with 0.2 µM final concentrations and Maxima® Hot Start Taq Polymerase in 2X Hot Start PCR buffer with 400 µM dNTPs (Thermo Scientific). No template control was added to all PCR reactions. The cycling parameters for first PCR were 15 min at 95°C, followed by 3 cycles of 30 s at 50°C, 60 s at 72°C and 30 s at 95°C, then 28 cycles of 30 s at 65°C, 60 s at 72°C and 30 s at 95°C with a final extension at 72°C for 10 min; and for second PCR: 15 min at 95°C, followed by 5 cycles of 30 s at 62°C, 60 s at 72°C and 30 s at 95°C, then 20 cycles of 60 s at 72°C, 30 s at 95°C and a final extension at 72°C for 10 min.  Amplicons of second PCR were purified with Agentcourt AMPureXP kit (Beckman Coulter Inc.) in plate format. Samples were eluted in Buffer EB (10mM Tris-Cl, pH 8.5; QIAGEN Inc.). DNA concentration of purified amplicons was measured using Appliskan fluorescence-based microplate reader (Thermo Scientific) and PicoGreen® dsDNA Quantification Reagent (Quant-iT dsDNA Broad Range Assay Kit, Invitrogen). The sequencing of the DNA library was performed on the Roche 454 FLX next generation sequencing platform. |
| ***Real-time quantitative PCR* (*q*PCR)** |
| Total bacterial concentration and three common bacteria of reproductive tract (*Enterobacteriaceae, Staphylococcus* sp., and *Corynebacterium* sp.) were additionally tested using real-time PCR method.  To generate plasmid standards for qPCR, plasmids containing amplified region of target bacteria were cloned using the pGEM-T Easy vector system (Promega, Madison, USA). Corresponding PCR amplicons were inserted into a separate plasmid vector and recombinant vector was transformed into chemically competent *E. coli* JM109 cells. Plasmids were purified with NucleoSpin Plasmid QuickPure kit according to manufacture instruction (Macherey-Nagel, Germany). Multiple dilutions of purified plasmids were quantified by spectrophotometry (NanoDrop ND-1000, USA) (13, 16). Quantification of target DNA was achieved by using serial tenfold dilution from 10^5^ to 10^1^ plasmid copies of the previously quantified plasmid standards.  Amplification and detection of DNA by real-time PCR was performed with a 7500 Fast Real-Time PCR System (Applied Biosystems Europe BV, Zug, Switzerland) using optical-grade 96-well plates. Triplicate sample analysis was routinely performed in a total volume of 25 µl using SYBR Green PCR Master Mix (Applied Biosystems). Each reaction included 5 µl of template DNA or water (no-template control), 12.5 µl of SYBR Green Master mix (Applied Biosystems, USA), 4 mM MgCl_2_ and the appropriate primers with concentration: *Enterobacteriaceae* (200 nM)*,* *Staphylococcus* sp. (250 nM) and *Corynebacterium* sp. (250 nM) (Table 1).  TaqMan assay PCR reaction was performed in a total volume of 25 µl using the TaqMan® Universal PCR Master Mix (Applied Biosystems, USA). Each reaction included 5µl of template DNA or water, 12.5 µl of TaqMan® Universal PCR Master Mix (Applied Biosystems, USA), 100 nM of each primer and 50 nM probe (Table 1).  The real-time PCR conditions consisted of an initial denaturation step 50°C for 2 min and 95°C for 10 min, continued with amplification step followed by 40 cycles consisting of denaturation at 95°C for 15 s, and annealing-elongation step at 60°C for 1 min. At the end of PCR assays dissociation curve analysis was performed to check for non-specific products and/or contamination. Standard curves were routinely performed for each real-time PCR run using serial dilutions of control plasmid DNA. Data were analyzed using the Sequence Detection Software version 1.6.3 (Applied Biosystems, USA). |
| ***Sequence processing and taxonomic assessment*** |
| The initial pre-trimmed data set was denoised using PyroNoise and UChime (“chimera.uchime” task in *de novo* mode) implemented in MOTHUR software 1.27.0. Only sequences longer than 150 bp were included for further processing. Operational taxonomic units (OTUs) were generated with the average neighbour hierarchical clustering algorithm with identity threshold of 97%. For additional denoising, OTUs with less than 5 sequences were removed. Reference sequences of aligned 16S rDNA were obtained from the SILVA rRNA database (17), while the taxonomic assignments were performed using Naive Bayesian classifier with a confidence cut-off of 90% (18). OTU naming was based on the lowest taxonomic level identified for OTUs based on SILVA database match followed by the value of this match (100 equals 100% match) and serial number in the OTU list generated for this study (the list was generated in random order). Additional taxonomic assignment for identifying species, to which OTUs potentially corresponded to, was performed using BLASTN against the NCBI nt database. The relative abundance values and sequence counts were all normalized. Principal coordinate analysis (PCoA) plots were generated using the software package PAST 3.20 (http://www.softpedia.com/get/Science-CAD/PAST.shtml), which allows analysis of high-throughput community sequencing data. |

**Supplementary Table S2** Overview of the semen and vaginal samples of the study subjects

| **Semen samples** | | | | | | | | |
| --- | --- | --- | --- | --- | --- | --- | --- | --- |
| **Study**  **samples (n=50)** | **Reference *** | | **Raw**  **semen samples *** | | **Washed sperm suspension** | **Incubated**  **sperm suspension** | | ***P*** |
| Sperm counts (millions/ml) | ≥ 20 | | 76.5 **±** 50.03 **^a,b^** | | 20.9 **±**  16.04 **^a,c^** | 3.7 **±**  0.78 **^c, b^** | | **^a^** <0.001  **^b^** <0.001  **^c^** <0.001 |
| Motility A+B (%) | ≥ 50 | | 69.2 **±** 12.43 **^d,e^** | | 79.3  **±**12.35 **^d^** | 79.3  **±**12.35 **^e^** | | **^d^** <0.001  **^e^** <0.001 |
| Sperm volume (ml) | ≥ 2.0 | | 3.8 **±** 1.06 | | - | - | | - |
| Round cells **(millions/ml) | ≤ 1 | | 2.32 ± 2.11 | | - | - | | - |
| Neutrophils  (millions/ml) *** | ≤ 0.2 ^&^ | | 0.15 ± 0.36 | | - | - | | - |
| **Vaginal samples** | | | | | | | | |
| **Morphotype** | | **Count of particular morphotype seen per field** | | | | | | |
|  |  | **0-1+** | | **2­-3+** | | | **4+** | |
| *Lactobacillus* spp. | | 8 | | 2 | | | 40 | |
| *Gardnerella/Bacteroides* | | 46 | | 0 | | | 14 | |
| *Mobiluncus* spp. | | 50 | | 0 | | | 0 | |
|  | | Number of subjects | | | | | | |
| Nugent score ♦ | | Score 0-3  (normal) | | Score 4-6  (intermediate) | | | Score 7-10  (bacterial vaginosis) | |
|  |  | 36 | | 6 | | | 8 | |
| Other elements | | - | | +/- | | | + | |
| Clue cells | | 38 | | 3 | | | 9 | |
| Leukocytes | | 28 | | | | | 12/10 ^#^ | |
| Other bacteria | | 21 | | 0 | | | 29 | |
| Candida | | 0 | | 0 | | | 2 | |

♦Score 0–3 (normal), score 4–6 (intermediat), score 7–10 (bacterial vaginosis)

* Reference values are valid for collected semen samples only (before washing)

** Include all round cells (epithelial cells, spermatogenesis cells, leukocytes). Round cells were present in 72.0% of raw samples.

*** Neutrophils were present in 20.0% of raw semen samples.

^&^ Punab *et al*., 2003

^#^ moderate / abundant

**Supplementary Table S3** Overview of general and reproductive health of female partners

| **Anthropometric and health parameters** | | **Per cent, mean ± SD** |
| --- | --- | --- |
| Age (years) |  | 33.4 ± 4.4 |
| Weight (kg) |  | 65.6 ± 11.8 |
| Length (cm) |  | 169.2 ± 5.3 |
| Menarche (years) |  | 13.3 ± 1.4 |
| Intimate hygiene | Vaginal rinse  Antibacterial soap  Intimate wash gel  Tampons | 2.0%  10%  16%  28% |
| Smoking | Never  In the past  Periodically  Currently | 54%  24%  6.0%  4.0% |
| Alcohol consumption | Never  1-4 times a year  1 x / month  2 x /month  1-2 x week  Daily | 8.0%  14%  24%  26%  16%  0.0% |
| Risk factors at work | Chemicals  Radiation  Molds  Humidity  Physical overload  Mental overload | 2.0%  0.0%  2.0%  2.0%  9.0%  18% |
| Physical activity and sport | No  Walking, jogging  Bicycling  Swimming  Aerobics  Skiing  Roller-skating  Ball games  Yoga  Dancing  Gym  Other | 22%  42%  20%  8.0%  8.0%  4.0%  4.0%  2.0%  8.0%  4.0%  4.0%  2.0% |
| Previous pregnancies  Mean number of previous pregnancies | | 60%  1.4 ± 1.3 |
| Spontaneous abortions |  | 20% |
| Extrauterine pregnancies |  | 14% |
| Artificial abortions |  | 24% |
| Previous deliveries |  | 34% |
| Investigations due to infertility |  | 92% |
| Previous treatments due to infertility | Hormonal  Intrauterine insemination (IUI)  *In Vitro* fertilization (IVF)  Intracytoplasmic sperm Injection (ICSI)  Frozen Embryo Transfer (FET)  Other | 28%  14%  60%  4.0%  30%  8.0% |
| Oral sex | Man to woman  Woman to man | 34%  24% |
| Anal sex |  | 4.0% |
| Sexual activity during last month | Daily  5-6 x / week  3-4 x / week  1-2 x / week  2-3 x / month  1 x / month  No sex during last month | 0.0%  0.0%  10%  54%  12%  4.0%  8.0% |
| Discomfort during sex | Never  Rarely  Less than half  Half  More than half  Always | 14%  58%  4.0%  6.0%  2.0%  4.0% |
| Genital tract infections in anamnesis ^1^ |  | 42% |
| Gynecological surgery |  | 76% |
| Chronic salpingitis |  | 10% |
| Missing or closed fallopian tubes |  | 48% |
| Endometriosis |  | 22% |
| Uterine myoma |  | 12% |
| Polycystic ovary syndrome |  | 10% |
| Success rate of current assisted reproductive technology procedure | Biochemically detected  Clinically detected | 36%  28% |
| Embryo quality | Grade 1  Grade 2  Grade 3 | 48%  50%  2.0% |

|  |  |  |  |  |  |
| --- | --- | --- | --- | --- | --- |

**Supplementary Table S4** Overview of general and reproductive health of male partners

| **Anthropometric and health parameters** | | **Per cent, mean ± SD** |
| --- | --- | --- |
| Age (years) |  | 37.1± 6.3 |
| Weight (kg) |  | 90.7 ± 12.4 |
| Length (cm) |  | 182.5 ± 6.7 |
| Smoking | Never  In the past  Periodically  Currently | 34%  28%  6.0%  18% |
| Alcohol consumption | Never  1-4 times a year  1 x / month  2 x / month  1-2 x / week  Daily | 6.0%  10%  12%  26%  34%  2.0% |
| Risk factors at work | Chemicals  Radiation  Molds  Humidity  Physical overload  Mental overload | 6.0%  0.0%  0.0%  2.0%  6.0%  14% |
| Physical activity and sport | No  Walking, jogging  Bicycling  Swimming  Aerobics  Skiing  Roller-skating  Ball games  Yoga  Dancing  Gym  Other | 20%  30%  22%  2.0%  2.0%  2.0%  2.0%  16%  0.0%  2.0%  6.0%  8.0% |
| Common children with current partner |  | 28% |
| Previously caused pregnancy |  | 20% |
| Complaints of urination | No  Frequent urination  Urgency  Weak urinary flow  Sensation of not emptying bladder  Pain  Blood in urine  Drip  Difficulty to start  Nocturia | 64%  6.0%  4.0%  0.0%  0.0%  2.0%  0.0%  6.0%  0.0%  16% |
| Pain or discomfort during last  month | No  In testes  In groin  In hypogastrium  In penis  In perineum  In sacrum  Uncertain | 58%  10%  2.0%  2.0%  2.0%  4.0%  16%  2.0% |
| **Genital tract infections in anamnesis** | | |
| Mycoplasmosis or ureaplasmosis  Genital herpes  Papillomavirus infection  Prostatitis  Candidiasis  Unknown infections | | 8%  2%  2%  4%  2%  8% |
| Surgery in genital tract region |  | 6.0% |
| Normal semen analysis |  | 72% |

**Supplementary Table S5** Relative abundance (%) of different bacterial phyla in microbial communities of different samples (mean±SD)

| **Phylum** | **Semen** | **Washed sperm** | **Incubated**  **sperm** | **IVF culture media** | **P** |
| --- | --- | --- | --- | --- | --- |
| *Firmicutes* | 79.7±24.4^a,b,c^ | 31.5±21.1^a^ | 5.3±1.8^b^ | 40.3±20.6^c^ | ^a^0.003  ^b^0.028  ^c^0.022 |
| *Bacteroidetes* | 13.9±12.4^d^ | 22.1±15.7^d^ | 25.0±29.3 | 12.8±2.7 | ^d^0.011 |
| *Proteobacteria* | 9.9±15^e,f^ | 38.4±31.5^e^ | 53.3±35.8 | 47.8±15.6^f^ | ^e^0.007  ^f^0.006 |
| *Actinobacteria* | 2.8±2.5 | 4.9±2.9 | 14.6±9.4 | 4.4±3.7 | NS |
| *Acidobacteria* | 0.006±0.01 | 0.0±0.0 | 0.0±0.0 | 0.0±0.0 | NS |
| *Fusobacteria* | 0.03±0.1 | 0.01±0.03 | 0.0±0.0 | 0.0±0.0 | NS |
| *Deinococcus-Thermus* | 0.03±0.02 | 0.36±0.8 | 0.6±0.8 | 0.2±0.2 | NS |
| *Verrucomicrobia* | 0.0±0.0 | 0.0±0.0 | 0.0±0.0 | 0.09±0.1 | NS |
| *Chloroflexi* | 0.002±0.008 | 0.0±0.0 | 0.0±0.0 | 0.0±0.0 | NS |
| *Synergistetes* | 0.005±0.02 | 0.0±0.0 | 0.0±0.0 | 0.0±0.0 | NS |
| *Cyanobacteria* | 0.006±0.02 | 0.0±0.0 | 0.0±0.0 | 0.0±0.0 | NS |
| *TM7* | 0.003±0.009 | 0.0±0.0 | 0.0±0.0 | 0.0±0.0 | NS |
| *Tenericutes* | 0.015±0.06 | 0.0±0.0 | 0.0±0.0 | 0.0±0.0 | NS |
| Unclassified  bacteria | 1.0±3.0 | 2.6±2.4 | 1.0±0.15 | 0.6±0.5 | NS |

**Supplementary Table S6** Relative abundance (%) of different bacterial classes in microbial communities of different samples (mean±SD)

| **Phylum** | **Class** | **Semen** | **Washed sperm** | **Incubated sperm** | **IVF culture media** | **P** |
| --- | --- | --- | --- | --- | --- | --- |
| *Firmicutes* | *Bacilli* | 72.9±30^a,b,c^ | 6.5±6.6^a^ | 0.7±0.1^b^ | 39±20.6^c^ | ^a^<0.001  ^b^0.028  ^c^0.006 |
|  | *Clostridia* | 6.5±14.3 | 24.8±26.8 | 4.4±1.8 | 1.35±1.9 | NS |
|  | *Erysipelotrichia* | 0.09±0.14 | 0.0±0.0 | 0.0±0.0 | 0.0±0.0 | NS |
| *Bacteroidetes* | *Bacteroidia* | 4.1±8.3^d^ | 13.3±13.7 | 22.4±30.2^d^ | 0.4±0.8 | ^d^0.028 |
|  | *Flavobacteriia* | 0.04±0.08 | 1.15±24.3 | 0±0 | 0.6±0.9 | NS |
| *Proteobacteria* | *Alphaproteobacteria* | 8.5±15^e^ | 21.8±24.3 | 45.7±34 | 40.8±14.8^e^ | ^e^0.011 |
|  | *Betaproteobacteria* | 0.14±0.2^f,g^ | 7.3±13.1^f^ | 2.0±1.6 | 1.8±1.1^g^ | ^f^0.027  ^g^0.003 |
|  | *Deltaproteobacteria* | 0.006±0.02 | 0.0±0.0 | 0.2±0.3 | 0.0±0.0 | NS |
|  | *Gammaproteobacteria* | 0.5±0.5^h^ | 5.3±10 | 1.4±1.2 | 5.1±1.0^h^ | ^h^0.003 |
|  | *Epsilonproteobacteria* | 0.6±1.5 | 3.75±4.7 | 2.3±3.2 | 0.0±0.0 | NS |
|  | *Sphingobacteria* | 1.6±3.3 | 3.5±4.0 | 0.5±0.7 | 5.0±2.5 | NS |
| *Actinobacteria* | *Actinobacteria* | 2.8±3.0 | 4.9±2.9 | 14.4±9.6 | 4.4±3.7 | NS |
| *Acidobacteria* | *Acidobacteria Gp 4* | 0.002±0.01 | 0.0±30 | 0.0±0.0 | 0.0±0.0 | NS |
| *Fusobacteria* | *Fusobacteria* | 0.03±0.1 | 0.01±0.03 | 0.0±0.0 | 0.0±0.0 | NS |
| *Deinococcus-Thermus* | *Deinococci* | 0.007±0.01 | 0.36±0.8 | 0.6±0.8 | 0.2±0.25 | NS |
| *Verrucomicrobia* | *Verrucomicrobia* | 0.0±0.0 | 0.0±0.0 | 0.0±0.0 | 0.005±0.1 | NS |
| *Chloroflexi* | *Chloroflexia* | 0.002±0.008 | 0.0±0.0 | 0.0±0.0 | 0.0±0.0 | NS |
| *Synergistetes* | *Synergistia* | 0.006±0.02 | 0.0±0.0 | 0.0±0.0 | 0.0±0.0 | NS |
| *Cyanobacteria* | *Cyanobacteria* | 0.03±0.06 | 0.0±0.0 | 2.1±3.0 | 0.05±0.1 | NS |
| *Candidate* | *TM7 genera Incertae*  *Sedis* | 0.002±0.002 | 0.0±0.0 | 0.0±0.0 | 0.0±0.0 | NS |
| *Tenericutes* | *Mollicutes* | 0.015±0.06 | 0.0±0.0 | 0.0±0.0 | 0.0±0.0 | NS |
| *Spirochaetes* | *Spirochaetia* | 0.0±0.0 | 0.007±0.01 | 0.0±0.0 | 0.0±0.0 | NS |
| *Unclassified* | *Unclassified* | 1.6±5.4 | 1.0±0.1 | 1.0±0.1 | 0.97±0.5 | NS |

**Supplementary Table S7** Relative abundance (%) of most frequent bacterial genera of microbial communities of different samples (mean±SD)

| **Class** | **Genus** | **Semen** | **Washed sperm** | **Incubated sperm** | **IVF culture solution** | **P** |
| --- | --- | --- | --- | --- | --- | --- |
| *Bacilli* | *Lactobacillus* | 73.3±27.2^a,b^ | 2.3±2.5^a^ | 0±0^b^ | 35.5±24.6 | ^a,b^<0.05 |
|  | *Staphylococcus* | 4.0±5.5 | 4.4±4.4 | 3.4±0.49 | 0.3±0.5 | NS |
|  | *Streptococcus* | 0.1±0.1 | 0.3±0.6 | 0±0 | 0±0 | NS |
|  | *Geobacillus* | 0.05±0.1^c^ | 0.07±0.1^d^ | 0.1±0.02 | 2.9±2.4^c,d^ | ^c,d^<0.05 |
| *Clostridia* | *Incertae sedis XI* | 4.5±13.4 | 22.3±24.2 | 0±0 | 0.7±1.4 | NS |
|  | *Peptonophilus* | 0.8±1.6 | 5.3±6.4 | 0±0 | 0±0 | NS |
|  | *Anaerococcus* | 0.2±0.3 | 0.6±0.7 | 0±0 | 0±0 | NS |
|  | *Blautia* | 0.4±0.5 | 0±0 | 0±0 | 0±0 | NS |
|  | *Finegoldia* | 0.3±0.5 | 0.7±0.9 | 0±0 | 0±0 | NS |
|  | *Fastidiosipila* | 0±0 | 0.06±0.1 | 0±0 | 0±0 | NS |
|  | *Sporacetigenium* | 0±0 | 0±0 | 2.7±3.9 | 0±0 | NS |
|  | *Thermoanaerobacterium* | 0.3±0.5 | 0±0 | 0±0 | 0.3±0.5 | NS |
| *Bacteroidia* | *Prevotella* | 3.9±7.1 | 9.1±11.5 | 24.5±30.4 | 0±0 | NS |
|  | *Porphyromonas* | 0.14±0.3 | 4.86±11.3 | 0±0 | 0±0 | NS |
|  | *Sediminibacterium* | 0.06±0.09^e^ | 2.3±3.6 | 0±0^f^ | 5.3±3.3^e,f^ | ^e^<0.05  ^f^<0.05 |
| *Flavobacteriia* | *Flavobacterium* | 0.02±0.08 | 2.0±3.0 | 0±0 | 0±0 | NS |
| *Alphaproteo-bacteria* | *Methylobacterium* | 0.2±0.5 | 0±0^g^ | 14.6±4.7^g^ | 0.13±0.2 | ^g^<0.05 |
|  | *Sphingobium* | 0.03±0.5 | 0.03±0.07 | 4.1±5.8 | 2.2±2.4 | NS |
|  | *Novosphingobium* | 0.1±0.2^h^ | 5.7±9.0 | 7.0±7.6^h^ | 4.6±3.9 | ^h^0.002^♦^ |
|  | *Sphingomonas* | 0.2±0.6 | 0.2±0.4 | 1.1±1.5 | 3.5±2.5 | NS |
|  | *Brevundimonas* | 0.02±0.04 | 0±0 | 0±0 | 0.35±0.4 | NS |
|  | *Devosia* | 0.003±0.01 | 0.8±1.7 | 0.3±0.04 | 0±0 | NS |
|  | *Mesorhizobium* | 0.8±1.5 | 0±0 | 0±0 | 0.3±0.5 | NS |
|  | *Phyllobacterium* | 1.7±3.0^i^ | 8.9±14.7 | 17.7±13.3 | 25±8.9^i^ | ^i^<0.05 |
| *Betaproteobacteria* | *Variovorax* | 0.07±0.02 | 0±0 | 0±0 | 0.4±0.6 | NS |
|  | *Pelomonas* | 0.06±0.1^j^ | 2.1±2.5 | 0.4±0.6 | 0.9±0.4^j^ | ^j^<0.05 |
| *Gammaproteobacteria* | *Acinetobacter* | 0.1±0.2 | 0.4±0.9 | 0±0 | 0.2±0.2 | NS |
|  | *Pseudomonas* | 0.1±0.1^k^ | 1.1±2.8^l^ | 0.7±0.6 | 3.5±2.1^k, l^ | ^k,l^<0.05 |
| *Epsilonproteobacteria* | *Campylobacter* | 0.9±2.2 | 4.5±5.3 | 2.3±3.2 | 0±0 | NS |
| *Sphingobacteria* | *Niabella* | 2.3±5.1 | 0±0 | 0±0 | 0±0 | NS |
| *Actinobacteria* | *Corynebacterium* | 1.2±1.3 | 0.2±0.1 | 11±13.3 | 0.6±1.3 | NS |
|  | *Micrococcus* | 0.8±1.2 | 0±0 | 1.9±2.6 | 0±0 | NS |
|  | *Propionibacterium* | 0.04±0.04 | 0±0 | 1.1±1.2 | 0.5±0.3 | NS |
|  | *Arthrobacter* | 0.6±1.8 | 1.0±1.1 | 0±0 | 0.4±0.7 | NS |
|  | *Rhodococcus* | 0.03±0.04 | 0±0 | 0±0 | 1.9±1.9 | NS |
|  | *Mobiluncus* | 0.08±0.24 | 0.4±0.8 | 0±0 | 0±0 | NS |
| *Negativicutes* | *Dialister* | 0.5±1.1 | 1.3±1.2 | 0±0 | 0.04±0.09 | NS |

p<0.05 (Dunn’s Method); ^♦^ - Kruskal-Wallis One-way analysis of variance
